# One-year outcomes of elderly acute cholecystitis patients by index treatment

**DOI:** 10.3389/fsurg.2025.1500700

**Published:** 2025-01-30

**Authors:** Núria Lluís, Celia Villodre, Lucía Guilabert, Isabel de Castro, Pedro Zapater, Belén Martínez, José R. Aparicio, Fèlix Lluís, Enrique de-Madaria

**Affiliations:** ^1^ Department of Surgery, Hospital of the University of Pennsylvania, Philadelphia, PA, United States; ^2^ Department of Surgery, Dr. Balmis General University Hospital, Alicante, Spain; ^3^ Alicante Institute of Health and Biomedical Research (ISABIAL), Alicante, Spain; ^4^ Department of Gastroenterology, Dr. Balmis General University Hospital, Alicante, Spain; ^5^ Department of Nursing, Dr. Balmis General University Hospital, Alicante, Spain; ^6^ Department of Pharmacology, Dr. Balmis General University Hospital, Alicante, Spain; ^7^ Department of Pharmacology, Miguel Hernández University, Elche, Spain; ^8^ IDIBE, CIBERehd, Alicante, Spain; ^9^ Department of Medicine, Miguel Hernández University, Elche, Spain

**Keywords:** cholecystectomy, laparoscopic, conservative treatment/methods, drainage/methods, endosonography/methods, propensity score, retrospective studies

## Abstract

**Background:**

Strategies for managing the elderly with acute cholecystitis need to be refined.

**Aims:**

To examine additional procedures, hospital readmissions, and outpatient visits in the year following the index admission.

**Patients and methods:**

Single-institution retrospective study of fifty consecutive patients aged ≥70 years admitted with acute cholecystitis. A propensity score matching analysis adjusted for demographic and clinical variables was carried out.

**Results:**

The one-year rates of additional procedures were 0%, 47.4%, and 72.7% for surgery, supportive care (SC), and percutaneous gallbladder drainage (PCGD), respectively. The one-year readmission rate was 0%, 15.8%, and 50% after these index procedures, respectively. After propensity score analysis, patients who received SC (55.6% vs. 0%, *P* = .03) or PCGD (77.8% vs. 0%, *P* = .002) had a higher rate of additional procedures compared to those who underwent surgery. Additionally, patients receiving PCGD had a higher readmission rate than those undergoing surgery (55.6% vs. 0%, *P* = .03). Nine patients who received SC and nine patients who received PCGD could have potentially undergone surgery during the index admission. This would have resulted in improved one-year outcomes.

**Conclusion:**

Cholecystectomy during the index hospitalization may provide better one-year outcomes than SC or PCGD in at least 50% of patients ≥70 years with acute cholecystitis.

## Introduction

Global life expectancy is on the rise, leading to a significant increase in the elderly population worldwide. Projections indicate that by 2050, the number of individuals aged 80 years or older will triple, reaching 426 million (1). Within this demographic shift, gallbladder and biliary tract diseases emerge as prominent concerns within the spectrum of digestive tract disorders. Notably, women and the elderly are particularly vulnerable to these conditions (2). In fact, the prevalence of gallstones among the elderly ranges between 14% and 23%, while it approaches 80% among nonagenarians (3).

It´s crucial to recognize that elderly individuals with biliary disorders might initially exhibit mild symptoms but can rapidly decompensate without proper treatment. Conditions such as acute cholecystitis pose a significant threat as they can rapidly escalate to systemic inflammatory response syndrome, sepsis, and death (4). While age itself may not directly correlate with the severity of acute cholecystitis, the presence of multiple comorbidities in elderly patients can significantly contribute to the progression of the disease (5, 6).

The first therapeutic option in elderly patients with acute cholecystitis is supportive care, consisting of intravenous hydration, analgesia and antibiotics (3, 7). Although supportive care is a common treatment option because it avoids the risk associated with anesthesia and shortens the hospital stay, it does not solve the problem and carries a high risk of recurrence (7).

Currently, percutaneous gallbladder drainage is the most widely used non-surgical procedure in elderly patients with acute cholecystitis (3). It has been argued that it could be a definitive treatment for acute cholecystitis in high-risk elderly patients (8). However, other studies advocate percutaneous drainage as a bridge to laparoscopic cholecystectomy in high-risk elderly patients (9), as well as in octogenarians with acute cholecystitis (10).

Early or delayed cholecystectomy (during index admission) is the treatment of choice for acute cholecystitis in patients fit for surgery (3). The Tokyo guidelines (5), the American Association of Surgery of Trauma (AAST) system (11), and the American Association of Anesthesiologists (ASA) can be used to stratify patients. The surgical treatment of acute cholecystitis in elderly people has paralleled the evolution of laparoscopic techniques in recent decades (4, 6, 10, 12–27).

Recently, endoscopic ultrasonography (EUS)-guided drainage of the gallbladder is being used as definitive therapy or as a bridge to delayed cholecystectomy in patients with acute cholecystitis unfit for emergency surgery (28–40). EUS-guided gallbladder drainage has also been combined with endoscopic retrograde cholangiopancreatography (ERCP) in patients with coexisting choledochal biliary stones (38).

Whichever procedure is used, long-term follow-up of elderly patients is needed (3). Many studies included patients with acute cholecystitis in their 60s, 70s and 80s, but the age mix precluded drawing decade-tailored conclusions (41). Therefore, one-year outcomes in patients aged 70 years and older with acute cholecystitis were specifically compared in this single-center study.

## Patients and methods

### Study design

This retrospective observational study examined a consecutive cohort of patients aged 70 years or older with acute cholecystitis (ICD-10 Codes: K81, K82) who were admitted between May 2021 and May 2022 at Doctor Balmis General University Hospital, Alicante, Spain. Patient care followed the established standard protocols of the hospital. Informed consent was not obtained from the patients due to the retrospective nature of the study. The study protocol adhered to the ethical guidelines of the 1975 Declaration of Helsinki (6th revision, 2008). This study was approved by the Research Ethics Committee of the Dr. Balmis Hospital (CEIm: PI2024-078). The study design and analysis complied with the STROCCS Reporting Guidelines for Cohort Studies (42).

### Exclusion criteria

Patients meeting any of the following criteria were excluded from the study: (a) history of prior cholecystitis episodes, ERCP, or previous cholecystostomy; (b) concurrent diagnosis of pancreatitis or any terminal illness with a life expectancy of less than one year; (c) acute cholecystitis diagnosed as a secondary condition, or in conjunction with acute pancreatitis, cholangitis, bile duct disorders, or gastrointestinal malignancy; (d) acute cholecystitis discovered incidentally during another surgical procedure, Mirizzi syndrome, or prior diagnosis of gallbladder cancer. Incidental gallbladder cancer was not an exclusion criterion. We intentionally did not include four patients who underwent EUS-guided gallbladder drainage during the index admission, as the small number of patients would have prevented a statistical analysis.

### Demographics, baseline characteristics, and diagnosis

In addition to demographic data and past medical and surgical history, Charlson comorbidity index (CCI) (43), ECOG and Karnofsky performance status were recorded. The ASA score was used to ascertain patient's surgical risk. The definitive diagnosis of acute cholecystitis was graded according to the Tokyo guidelines. Duration of symptoms >72 h prior to admission was selected to categorize patients as it defines grade II in the Tokyo guidelines (5). Given that the Tokyo guidelines flowchart adds the coexistence of jaundice (total bilirubin >2 mg/dl) as one of the negative predictive factors in grade III acute cholecystitis, this laboratory value at admission was also selected as threshold to categorize patients (44). Laboratory values at admission used to define grades II and III in the Tokyo guidelines (i.e., creatinine >2.0 mg/dl, INR >1.5, platelet count <100,000/mm^3^, and white blood cell count >18,000/mm^3^) were selected as thresholds to categorize patients (5).

### Index treatment modalities

Supportive care *c*onsisted of intravenous hydration, analgesia utilizing acetaminophen or non-steroidal anti-inflammatory drugs, and administration of antibiotics (3). Percutaneous gallbladder drainage was carried out through either transperitoneal or transhepatic routes, accompanied by a radiological assessment prior to drain removal. In instances where surgery was deemed necessary, laparoscopic cholecystectomy was the preferred approach in most cases.

### Follow-up

Additional procedures, hospital readmissions due to acute cholecystitis-related complications, and outpatient encounters throughout the initial year following the index admission were recorded. Additional procedures included delayed cholecystectomy, percutaneous or endoscopy-guided gallbladder drainage procedures, and ERCP performed to remove stones or sludge from the common bile duct. Readmission episodes attributed to acute cholecystitis complications, encompassing recurrence, cholangitis, liver abscesses, common bile duct stones, and biliary colic were analyzed. Throughout the one-year outpatient follow-up, both planned appointments and unplanned encounters were documented. The latter encompassed instances where patients presented with complaints or symptoms pertaining to acute cholecystitis.

### Endpoints

The primary endpoints of the study were defined as follows: (a) additional procedures within the first year; (b) hospital readmissions within the first year attributable to biliary causes, and (c) outpatient encounters over the course of the first year, including both planned appointments and unscheduled visits.

### Data collection

Anonymized data was collected and managed using REDCap tools (REDCap®, Research Electronic Data Capture, University of Vanderbilt, Nashville, Tennessee, US) hosted at *Asociación Española de Gastroenterología* (AEG; https://www.redcap.aegastro.es) (45).

### Analysis of data

Descriptive statistics was used to analyze the demographic and baseline characteristics of the patients. Quantitative variables were presented as median and interquartile range (IQR), while categorical variables were expressed as absolute and relative frequencies. Group comparisons were performed using the Chi-square test or Fisher's exact test for categorical data, the *T*-test for parametric quantitative data, and the Mann–Whitney *U* test for quantitative non-parametric data. Although propensity score matching analysis has been used primarily for the comparison of two groups of subjects in observational studies, it has recently been considered for the analysis of more than two groups (46). However, due to the small number of subjects in this study, it was not possible to use it for more than two groups. Surgery was designated as the reference group for comparisons. Propensity score matching (1:1 optimal match) was performed using the MatchIt package for R software. The propensity score was determined via logistic regression, incorporating demographic and pre-procedure characteristics to mitigate the impact of selection bias, with a 0.1-caliper width employed. The procedure's impact was assessed by comparing outcomes (medians for continuous data, proportions for dichotomous data) following matching (47). Survival curves were generated utilizing the Kaplan–Meier method and subsequently compared using the log-rank test. *P* values of less than.05 were considered statistically significant. All analyses were performed using RStudio, version 1.2.5001 (Integrated Development for R. RStudio, Inc., Boston, MA, USA).

## Results

### Baseline population characteristics

A cohort of 50 patients diagnosed with acute cholecystitis were included in the study. Among them, 21 patients were septuagenarians, while 29 were octogenarians or older. During the index admission, cholecystectomy was performed on nine patients (18%), while 19 patients (38%) received supportive care. Additionally, percutaneous gallbladder drainage was carried out in 22 patients (44%).

Patients who received supportive care exhibited higher ASA scores (ASA 1, 21.1%; ASA 2, 63.2%; ASA 3, 15.8%) compared to those who underwent cholecystectomy (ASA 1, 77.8%; ASA 2, 22.2%) (*P* = .02) (Table 1). Patients who underwent percutaneous gallbladder drainage exhibited higher Charlson Comorbidity Index scores (7.5 [4.3–9.8] vs. 5 [4–5], *P* = .04), poorer ECOG performance status (*P* = .04), and elevated ASA scores (ASA 1, 18.2%; ASA 2, 63.6%; ASA 3, 18.2%) when compared to patients who underwent cholecystectomy (*P* = .01) (Table 2).

**Table 1:** Demographic and preoperative characteristics of septuagenarians and octogenarians undergoing surgery or supportive care for acute cholecystitis.

	Baseline population			After propensity score matching	
	Surgery *n* = 9	Supportive care *n* = 19	*P* value	Supportive care *n* = 9	*P* value
Age, years, median (IQR)	80 (75–83)	80 (74–82)	1.0	75 (72–80)	.27
Sex, *n* (%)			.68		1.0
Male	4 (44.4)	11 (57.9)		5 (55.6)	
Female	5 (55.6)	8 (42.1)		4 (44.4)	
Body mass index, BMI, kg/m^2^, median (IQR)	27.3 (27.0–32.9)	28.4 (26.9–29.7)	.71	28.6 (26.4–30.6)	
Tobacco use, *n* (%)			.19		.22
No	8 (88.9)	10 (52.6)		5 (55.6)	
Yes, former smoker	1 (11.1)	4 (21.1)		2 (22.2)	
Yes, current smoker	0	5 (26.3)		2 (22.2)	
Alcohol use, *n* (%)			.68		.21
No	9 (100)	15 (78.9)		6 (66.7)	
Yes, occasionally	0	1 (5.3)		0	
Yes, chronic alcoholism	0	3 (15.8)		3 (33.3)	
Previous abdominal surgical procedure, *n* (%)	2 (22.2)	7 (36.8)	.73	4 (44.4)	.62
Medication, *n* (%)					
Chronic steroid use	0	1 (5.3)	1.0	0	
Anticoagulant use	1 (11.1)	5 (26.3)	.67	2 (22.2)	1.0
Antiplatelet use	2 (22.2)	7 (36.8)	.73	3 (33.3)	1.0
Charlson Comorbidity Index, median (IQR)					
Score	5 (4–5)	5 (4.5–6)	.17	5 (4–6)	.52
Estimated 10-year survival	21 (21–53)	21 (2–37)	.20	21 (2–53)	.55
ECOG performance status, *n* (%)			.09		.22
0	1 (11.1)	0		0	
1	6 (66.7)	11 (57.9)		7 (77.8)	
2	0	6 (31.6)		2 (22.2)	
3	2 (22.2)	2 (10.5)		0	
Karnofsky performance status, median (IQR)	80 (80–80)	70 (60–80)	.14	80 (70–80)	.41
ASA score, *n* (%)			**.** **02**		.33
II	7 (77.8)	4 (21.1)		4 (44.4)	
III	2 (22.2)	12 (63.2)		5 (55.6)	
IV	0	3 (15.8)		0	
Time symptoms onset—admission					
Days, median (IQR)	3 (1–4)	2 (1–4)	.65	1 (1–2)	.42
>3 days, *n* (%)	3 (33.3)	6 (31.6)	1.0	2	1.0
Labs at admission, *n* (%)					
Total bilirubin >2.0 mg/ml	0	5 (26.3)	.14	2	.47
Creatinine >2.0 mg/dl	0	2 (10.5)	1.0	1	1.0
White blood cell count >18.0 × 10^3^	2 (22.2)	0	.09	0	.47
INR >1.5	0	3 (15.8)	.53	1	1.0
Platelet count <100 × 10^3^	0	3 (15.8)	.53	1	1.0
Tokyo grading of acute cholecystitis, *n* (%)			.43		1.0
I (mild)	3 (33.3)	10 (52.6)		4 (44.4)	
II (moderate)	6 (66.7)	9 (47.4)		5 (55.6)	
III (severe)	0	0		0	

Values with a *P* < .05 are shown in bold.

**Table 2:** Demographic and preoperative characteristics of septuagenarians and octogenarians undergoing surgery or percutaneous gallbladder drainage for acute cholecystitis.

	Baseline population			After propensity score matching	
	Surgery *n* = 9	Percutaneous gallbladder drainage, *n* = 19	*P* value	Percutaneous gallbladder drainage, *n* = 9	*P* value
Age, years, median (IQR)	80 (75–83)	81 (76.2–85.8)	.36	81 (77–85)	.48
Sex, *n* (%)			.28		.64
Male	4 (44.4)	16 (72.7)		6 (66.7)	
Female	5 (55.6)	6 (27.3)		3 (33.3)	
Body mass index, BMI, kg/m^2^, median (IQR)	27.3 (27.0–32.9)	29.3 (26.6–31.0)	1.0	29.3 (29–30)	
Tobacco use, *n* (%)			.38		1.0
No	8 (88.9)	15 (68.2)		8 (88.9)	
Yes, former smoker	1 (11.1)	7 (31.8)		1 (11.1)	
Yes, current smoker	0	0		0	
Alcohol use, *n* (%)			.67		—
No	9 (100)	18 (81.8)		9 (100)	
Yes, occasionally	0	3 (13.6)		0	
Yes, chronic alcoholism	0	1 (4.5)		0	
Previous abdominal surgical procedure, *n* (%)	2 (22.2)	6 (27.3)	1.0	2 (22.2)	1.0
Medication, *n* (%)					
Chronic steroid use	0	1 (4.5)	1.0	0	
Anticoagulant use	1 (11.1)	9 (40.9)	.23	3 (33.3)	.57
Antiplatelet use	2 (22.2)	6 (27.3)	1.0	3 (33.3)	1.0
Charlson Comorbidity Index, median (IQR)					
Score	5 (4–5)	7.5 (4.3–9.8)	.**04**	5 (4–8)	.53
Estimated 10-year survival	21 (21–53)	0 (0–45)	.06	21 (0- 53)	.62
ECOG performance status, *n* (%)			.**04**		1.0
0	1 (11.1)	1 (4.5)		1 (11.1)	
1	6 (66.7)	7 (31.8)		6 (66.7)	
2	0	10 (45.5)		0	
3	2 (22.2)	2 (9.1)		2 (22.2)	
4	0	2 (9.1)		0	
Karnofsky performance status, median (IQR)	80 (80–80)	60 (52.5–70)	.06	70 (70–80)	.42
ASA score, *n* (%)			.**01**		.15
II	7 (77.8)	4 (18.2)		3 (33.3)	
III	2 (22.2)	14 (63.6)		6 (66.7)	
IV	0	4 (18.2)		0	
Time symptoms onset—admission					
Days, median (IQR)	3 (1–4)	2.5 (1–4)	1.0	2 (1–4)	1.0
>3 days, *n* (%)	3 (33.3)	7 (31.8)	1.0	3 (33.3)	1.0
Labs at admission, *n* (%)					
Total bilirubin >2.0 mg/ml	0	2 (9.1)	1.0	2 (22.2)	.47
Creatinine >2.0 mg/dl	0	2 (9.1)	1.0	0	—
White blood cell count >18.0 × 10^3^	2 (22.2)	8 (42.1)	.88	3 (33.3)	1.0
INR >1.5	0	2 (9.1)	.88	1 (11.1)	1.0
Platelet count <100 × 10^3^	0	0	—	0	—
Tokyo grading of acute cholecystitis, *n* (%)			.28		1.0
I (mild)	3 (33.3)	2 (9.1)		2 (22.2)	
II (moderate)	6 (66.7)	17 (77.3)		6 (66.7)	
III (severe)	0	3 (13.6)		1 (11.1)	

Values with a *P* < .05 are shown in bold.

### Outcomes in the baseline population

**Figure 1:**
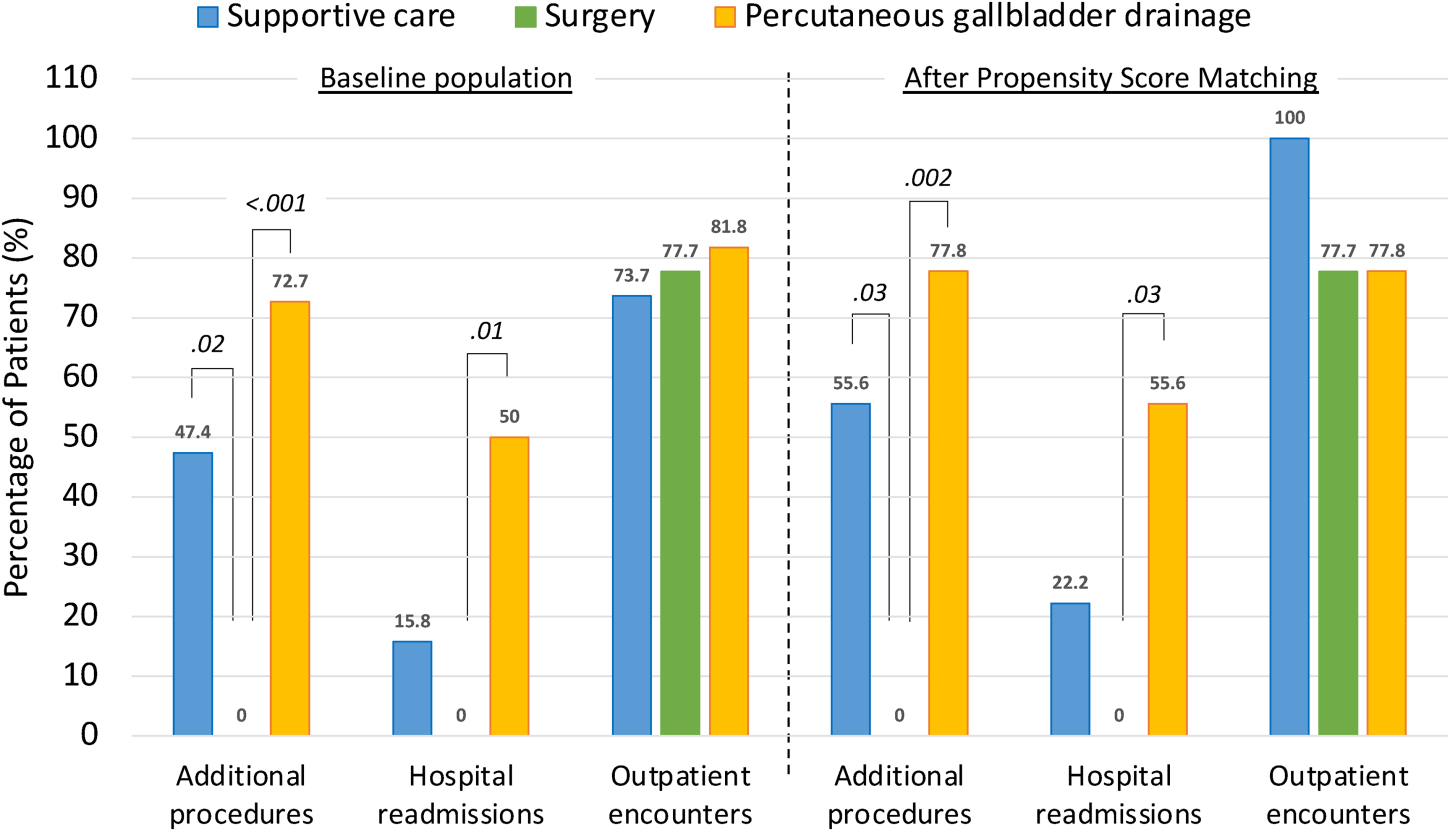
One-year outcomes of septuagenarians and octogenarians with acute cholecystitis undergoing either supportive care, surgery or percutaneous gallbladder drainage at index admission. Data labels indicate the percentage of patients who met a specific outcome after each index treatment modality, both in the baseline population (left panel) and after propensity score matching analysis (right panel). *P*-values *(in Italic)* were obtained considering surgery as reference.

A greater proportion of patients who received supportive care (47.4%) required additional procedures within one year compared to those who underwent cholecystectomy (0%) (*P* = .02) (Table 3, Figure 1). Furthermore, patients who received supportive care during their index admission required a higher number of additional procedures compared to those who underwent surgery (0 [0–1] vs. 0 [0–0], *P* = .02) (Table 3).

**Table 3:** One-year outcomes of septuagenarians and octogenarians with acute cholecystitis undergoing surgery or supportive care.

	Baseline population			After propensity score matching	
	Surgery *n* = 9	Supportive care *n* = 19	*P* value	Supportive care *n* = 9	*P* value
Additional procedures					
Patients, *n* (%)	0	9 (47.4)	.**02**	5 (55.6)	.**03**
Procedures, median (IQR)	0 (0–0)	0 (0–1)	.**02**	1 (0–1)	.**01**
Hospital readmissions					
Patients, *n* (%)	0	3 (15.8)	.53	2 (22.2)	.47
Readmissions, median (IQR)	0 (0–0)	0 (0–0)	.23	0 (0–0)	.17
Outpatient encounters					
Patients, *n* (%)	7 (77.8)	14 (73.7)	1.0	9 (100)	.45
Encounters/patient, median (IQR)	1 (1–1)	1 (0.5–2)	.25	2 (1–3)	.**01**

*P* values are vs. surgery.

Values with a *P* < .05 are shown in bold.

Similarly, significantly more patients undergoing percutaneous gallbladder drainage required additional procedures within one year (72.7% vs. 0%, *p* < 0.001) and experienced 1-year readmissions (50% vs. 0%, *p* = .01) compared to surgery (Table 4, Figure 1). In addition, the number of additional procedures (1 [0.25–1.75] vs. 0 [0–0], *P* < .001) and readmissions (0.5 [0–1] vs. 0 [0–0], *P* = .01) was significantly higher in patients who had percutaneous gallbladder drainage compared to those who had surgery during the index admission (Table 4).

**Table 4:** One-year outcomes of septuagenarians and octogenarians undergoing surgery or percutaneous gallbladder drainage for acute cholecystitis.

	Baseline population			After propensity score matching	
	Surgery *n* = 9	Percutaneous gallbladder drainage, *n* = 22	*P* value	Percutaneous gallbladder drainage, *n* = 9	*P* value
Additional procedures					
Patients, *n* (%)	0	16 (72.7)	**<**.**001**	7 (77.8)	.**002**
Procedures, median (IQR)	0 (0–0)	1 (0.25–1.75)	**<**.**001**	1 (1–2)	.**002**
Hospital readmissions					
Patients, *n* (%)	0	11 (50.0)	.**01**	5 (55.6)	.**03**
Readmissions, median (IQR)	0 (0–0)	0.5 (0–1)	.**01**	1 (0–2)	.**01**
Outpatient encounters					
Patients, *n* (%)	7 (77.8)	18 (81.8)	1.0	7 (77.8)	1.0
Encounters/patient, median (IQR)	1 (1–1)	2 (1–3)	.06	2 (1–3)n	.15

*P* values are vs. surgery.

Values with a *P* < .05 are shown in bold.

Additional procedures performed and reasons for readmission by treatment modality are summarized in Supplementary Table 1. In the supportive care group, nine of the 19 patients required a total of 14 additional procedures. Of these, three patients required six readmissions, all due to recurrence of acute cholecystitis. In addition, a total of 26 additional procedures were required in 16 of the 22 patients who underwent percutaneous gallbladder drainage. Of these patients, 11 required 15 readmissions, primarily for recurrent acute cholecystitis.

**Figure 2:**
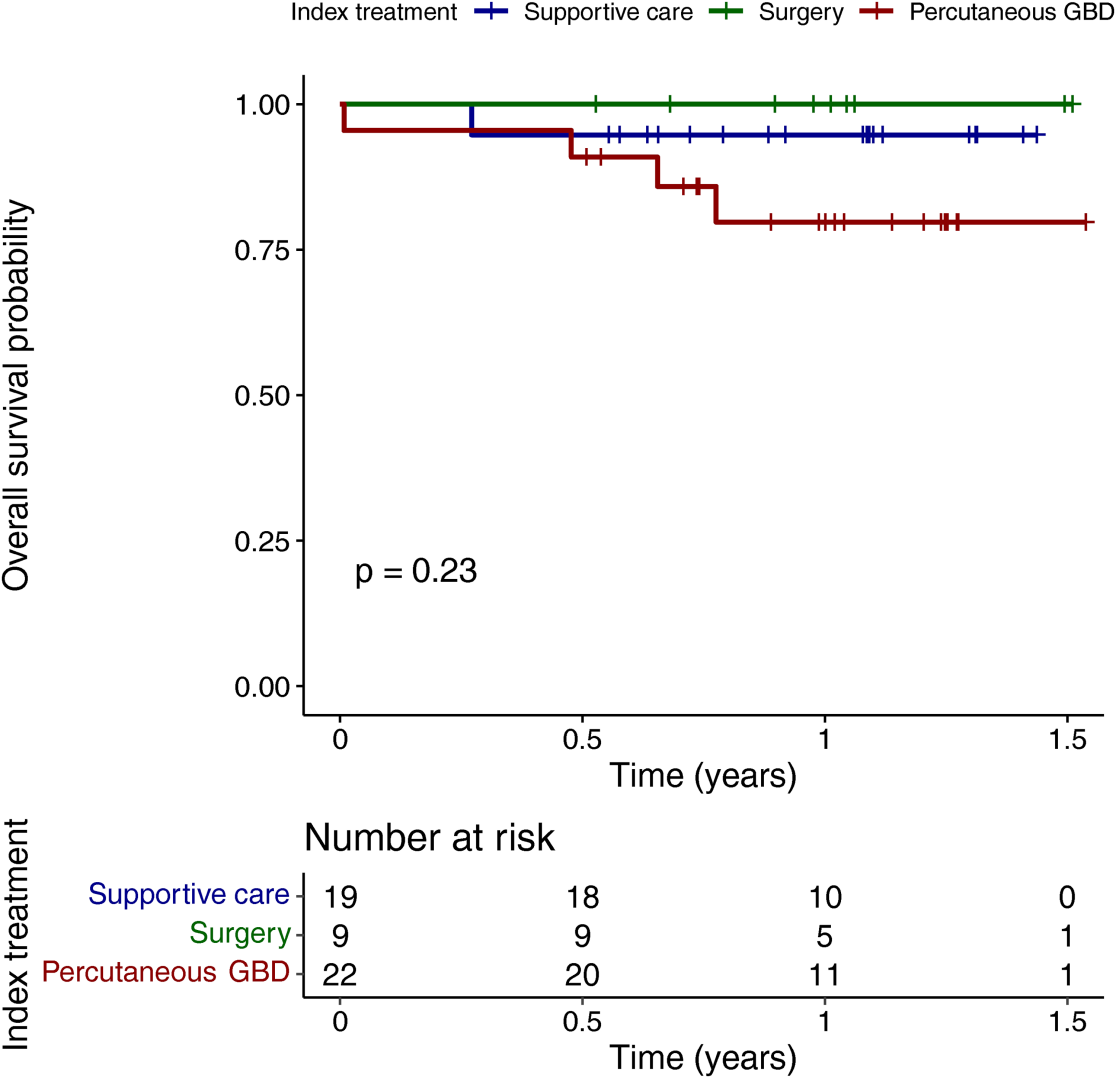
Overall survival of septuagenarians and octogenarians with acute cholecystitis by index treatment modality. GBD, gallbladder drainage.

One patient receiving supportive care and four patients undergoing percutaneous gallbladder drainage died during the follow-up period. Survival analysis was performed by index treatment modality within the baseline population. No survival differences were noted among the three index treatment modalities (Figure 2).

### Outcomes after propensity score matching analysis

After propensity score matching, there were no demographic or preoperative differences between the groups (Tables 1, 2). A higher proportion of supportive care patients required additional procedures within one year compared to surgery patients (55.6% vs. 0%, *P* = .03) (Table 3, Figure 1). In addition, the median number of additional procedures (1 [0–1] vs. 0 [0–0], *P* = .01) and outpatient encounters (2 [1–3] vs. 1 [1–1], *P* = .01) was significantly higher in patients who received supportive care compared to those who underwent surgery (Table 3).

Similarly, patients undergoing percutaneous gallbladder drainage were significantly more likely to require additional procedures within one year (77.8% vs. 0%, *P* = .002) and to be readmitted within one year (55.6% vs. 0%, *P* = .03) than patients undergoing surgery (Table 4, Figure 1). In addition, median numbers of additional procedures (1 [1–2] vs. 0 [0–0], *P* = .002) and rehospitalizations (1 [0–2] vs. 0 [0–0], *P* = .01) were significantly higher in patients undergoing percutaneous gallbladder drainage than in those undergoing cholecystectomy (Table 4).

After propensity score matching, the specific types of additional procedures performed and reasons for 1-year readmission attributed to acute cholecystitis-related complications are shown in Supplementary Table 1. A total of seven additional procedures were required in five patients who received supportive care. Additionally, two patients required four readmissions, all related to recurring acute cholecystitis. Among those who underwent percutaneous gallbladder drainage during the index admission, seven patients required a total of 12 additional procedures, with five patients requiring 10 readmissions for recurrent acute cholecystitis and other reasons.

A laparoscopic approach was used in all patients who underwent cholecystectomy during the index admission. In contrast, two patients who had a delayed cholecystectomy after supportive care or percutaneous gallbladder drainage required an open approach or conversion to an open approach (Supplementary Table 2). No cases of common bile duct injury or need for reoperation were observed in either group.

## Discussion

In summary, no additional procedures or readmissions were required in septuagenarians and octogenarians who underwent cholecystectomy during the index admission. In contrast, subsequent procedures and readmissions occurred in patients who received supportive care or underwent percutaneous gallbladder drainage. Based on the present study data, nine patients receiving supportive care and another nine patients undergoing percutaneous gallbladder drainage may have benefited from cholecystectomy during the index admission. Using this strategy, 27 of the 50 septuagenarians and octogenarians (54% of the baseline population) would have been eligible for surgery during their initial hospital admission.

A 4.4% rate of additional 30-day procedures was observed in a comprehensive Danish registry of more than 4,000 patients treated for acute cholecystitis, primarily by laparoscopic cholecystectomy (48). The majority of these were ERCP. The remainder were related to various surgical complications. In the Danish cohort, advanced age emerged as a notable determinant of the increased risk of needing additional procedures within 30 days. In contrast, the present study sought to determine whether additional procedures occurred within the first year following treatment. Of note, none of our elderly patients who underwent cholecystectomy had a need for additional procedures during the subsequent year. In contrast, the results obtained after propensity score matching showed that more than half of our patients who received supportive care (55.6%) and about three quarters of our patients who underwent percutaneous gallbladder drainage (77.8%) required subsequent procedures.

Planned readmissions, which occurred for scheduled procedures such as delayed cholecystectomy, endoscopic therapies, or interventional radiology procedures, were excluded from the readmission count within the first year. Unplanned readmissions included episodes of recurrent acute cholecystitis, as well as cases of acute cholangitis, liver abscess, common bile duct stone, and surgical site infection. Only the unplanned readmissions within the first year were included. In the present study, 1-year readmission rates varied significantly by treatment modality. Specifically, the rates were 0%, 15.8%, and 50% for surgery, supportive care, and percutaneous gallbladder drainage, respectively. These findings are consistent with those of a 5-year randomized controlled trial of 142 high-risk patients (APACHE II score ≥7). The trial demonstrated a significantly higher incidence of 30-day major complications, surgical and radiological re-interventions, readmissions, and emergency department visits after percutaneous gallbladder drainage compared to laparoscopic cholecystectomy (49). On the other hand, a comprehensive analysis of Medicare claims data from 1996 to 2005 emphasized the importance of performing a cholecystectomy during the initial hospitalization of elderly patients with acute cholecystitis. This approach was advocated to reduce risk of recurrent cholecystitis, multiple readmissions, and associated costs (50). Of note, differences in study design, patient demographics, hospital environment, and lack of propensity score matching analysis may contribute to the observed differences in readmission rates across previous studies (Supplementary Table 3).

A 100% 1-year survival rate was observed among those who underwent surgery in our cohort of patients. No statistically significant differences were observed among those who opted for alternative therapeutic modalities, especially after percutaneous gallbladder drainage. This may be due to the relatively small sample size. A 1-year survival rate of 82.2% after percutaneous cholecystostomy was reported in a study of 73 patients with acute cholecystitis in China with a median age of 82 years (8). Similarly, in Spain, a study of 113 octogenarian patients showed a 1-year survival rate of 86% after both emergency and delayed (beyond 48 h after stabilization) cholecystectomy (14). The Medicare study found a 1-year survival rate of 85% after cholecystectomy. This compares to 80.6% without definitive therapy (50).

The results of these studies highlight the importance of refinement of selection criteria for older patients with acute cholecystitis to be considered for surgery during their index hospitalization. This may spare them unnecessary, costly, and risky procedures, reduce the probability of readmissions and outpatient visits, and significantly improve their overall quality of life. The goal of treatment is to improve the patient's quality of life with minimal physiologic stress, ideally allowing the patient to return to previous levels of performance. Lifestyle considerations are an increasingly important part of the decision-making process for medical interventions in the elderly. Percutaneous gallbladder drainage, especially if it requires frequent catheter changes, can significantly impact quality of life.

### Limitations

There are several limitations to our study. First, it included retrospective data from a single center with a relatively small patient cohort. Second, only four patients underwent EUS-guided gallbladder drainage during the study period. Therefore, they could not be included in the statistical analysis. Third, a notable gap in this study is the lack of data on patient-reported outcomes, particularly quality of life. Research has shown that acute cholecystitis in the elderly population can have a profound impact on their perception of quality of life. Therefore, this aspect is of particular importance (2, 8, 32, 35, 51–53). Finally, statistical comparisons between variables were limited by the heterogeneous data collection for cholecystectomies performed during index and delayed admissions. Despite this, all index cholecystectomies were performed laparoscopically, whereas some delayed cholecystectomies began as open procedures or were converted to open. Notably, intense fibrosis around the common bile duct or adjacent viscera was observed in more than half of the delayed cholecystectomies.

## Conclusion

This study suggests that the decision to proceed with surgery during the index admission may result in superior one-year outcomes compared with supportive care or percutaneous gallbladder drainage in at least 50% of septuagenarians and octogenarians who are diagnosed with acute cholecystitis. Additional prospective data are needed to determine whether healthcare providers responsible for initial triage should identify older patients suitable for surgery during the index admission and prioritize this approach within acute care surgical protocols.

## Data Availability

The raw data supporting the conclusions of this article will be made available by the authors, without undue reservation.
