# Supplementary material for: One-year outcomes of elderly acute cholecystitis patients by index treatment
Source: Front Surg. 2025 Jan 30;12:1500700. doi: 10.3389/fsurg.2025.1500700 (PMC11821576; doi:10.3389/fsurg.2025.1500700)
Supplement: Supplementary file 1 [file Table1.docx]

**SUPPLEMENTARY MATERIAL**

**One-year outcomes of elderly acute cholecystitis patients by index treatment**

**Núria Lluís^1,#,*^, Celia Villodre^2,6,#^, Lucía Guilabert^3,6^, Isabel de Castro^4,6^, Pedro Zapater^5,6,7,9^, Belén Martínez^3,6^, José R Aparicio^3,6^, Fèlix Lluís^6^, Enrique de-Madaria^3,6,8^**

^1^Department of Surgery, Hospital of the University of Pennsylvania, Philadelphia, PA, USA.

Departments of ^2^Surgery, ^3^Gastroenterology, ^4^Nursing, and ^5^Pharmacology, Dr. Balmis General University Hospital, Alicante, Spain

^6^Alicante Institute of Health and Biomedical Research (ISABIAL), Alicante, Spain.

^7^Departments of Pharmacology, and ^8^Clinical Medicine, Miguel Hernández University, Elche, Spain

^9^IDIBE, CIBERehd, Alicante, Spain.

^#^ Contributed equally

**Index**

**Supplementary Table 1**. One-year additional procedures and reasons for 1-year readmission due to acute cholecystitis-related complications according to index treatment modality*

**Supplementary Table 2**. Perioperative data of patients who underwent cholecystectomy during the index admission (upfront surgery) or delayed cholecystectomy (delayed surgery).

**Supplementary Table 3**. Readmissions due to biliary-related reasons or recurrence in patients with acute cholecystitis. A literature reviews.

**Supplementary Table 1**. One-year additional procedures and reasons for 1-year readmission due to acute cholecystitis-related complications according to index treatment modality*

|  | **Baseline population** | | | **After propensity score matching** | |
| --- | --- | --- | --- | --- | --- |
|  | Surgery, n=9 | Supportive care, n=19 | Percutaneous gallbladder drainage, n=22 | Supportive care, n=9 | Percutaneous gallbladder drainage, n=9 |
| **1-year additional procedures, n** | 0 patients | 9 patients | 16 patients | 5 patients | 7 patients |
| Surgery (delayed cholecystectomy) |  | 4 | 4 | 2 | 4 |
| Percutaneous gallbladder drainage |  | 3 | 4 | 2 | 1 |
| Endoscopic gallbladder drainage |  | 1 | 8 |  | 2 |
| ERCP/ bile duct stone, sludge removal |  | 5 | 10 | 2 | 5 |
| Other |  | 1*^¶^* |  | 1*^¶^* |  |
| Total additional procedures | 0 | 14 | 26 | 7 | 12 |
| **Reasons for 1-year readmission, n** | 0 patients | 3 patients | 11 patients | 2 patients | 5 patients |
| Recurrence of acute cholecystitis |  | 6 | 8 | 4 | 6 |
| Acute cholangitis |  |  | 2 |  | 1 |
| Liver abscess |  |  | 1 |  | 1 |
| Common bile duct stones |  |  | 2 |  | 1 |
| Surgical site infection (deep) |  |  | 2 |  | 1 |
| Total readmissions | 0 | 6 | 15 | 4 | 10 |

**, only patients who required additional procedures or readmissions are included; ^¶^, percutaneous drainage of intraabdominal collection*

**Supplementary Table 2**. Perioperative data of patients who underwent cholecystectomy during the index admission (upfront surgery) or delayed cholecystectomy (delayed surgery).

|  | **Upfront surgery, n=9** | **Delayed surgery, n=8*** |
| --- | --- | --- |
| Surgical approach, n (%) |  |  |
| Open | 0 | 1 (12.5) |
| Laparoscopic | 9 (100) | 5 (62.5) |
| Conversion to open | 0 | 1 (12.5) |
| Missing** | 0 | 1 (12.5) |
| Upfront surgery |  |  |
| CBD exploration, n | 0 | - |
| T-tube placement, n | 0 | - |
| Operative time, min (IQR) | 89 (73 – 109) | - |
| Estimated blood loss, mL (IQR) | 100 (100 – 100) | - |
| Intraoperative pRBC transfusion, patients, n | 0 | - |
| Intraoperative adverse events, n | 0 | - |
| Surgical drain placement, n (%) | 4 (44.4) | - |
| CBD injury, n | 0 | - |
| Reoperation | 0 | - |
| Delayed cholecystectomy, patients, n (%) |  |  |
| Intense fibrosis/adherence to CBD or surrounding viscera | - | 5 (52.5) |
| CBD injury | - | 0 |
| Reoperation | - | 0 |
| Presence of incidental gallbladder cancer on the specimen | - | 0 |

**, after supportive care, n=4; after percutaneous gallbladder drainage, n=4*

***, managed at an outside center*

**Supplementary Table 3**. Readmissions due to biliary-related reasons or recurrence in patients with acute cholecystitis. A literature reviews.

| **Index treatment** | **Author** | **Year** | **Period of study** | **Source** | **Age threshold** | **Follow-up period** | **Readmission rate (%)** |
| --- | --- | --- | --- | --- | --- | --- | --- |
| PGBD vs Surgery | Loozen (1) | 2018 | 2011 – 2016 | RCT | All ages | 1-year | 65 % vs 8% |
|  | Sanaiha (2) | 2020 | 2010 – 2015 | NRD | ≥ 65 years | 90-day | 41.3% vs 6.4% |
|  | Garcés-Albir (3) | 2020 | 2005 – 2016 | Single center | ≥ 70 years | 30-day | 34.3% vs 10.7% |
|  | Fleming (4) | 2019 | 2013 – 2014 | NRD | All ages | 30-day | 33% vs 14.8% |
| SC vs Surgery | Kivivuori (5) | 2023 | 2017 – 2019 | Single center | ≥ 75 years | 1-year | 44% vs 8% |
| SC vs PGBD | Turiño (6) | 2019 | 2006 – 2015 | Single center | All ages | 1.6-years | 32.7% vs 38.6% |
| SC vs PGBD vs Surgery | Wiggins (7) | 2018 | 1997 – 2012 | HESD | ≥ 80 years | Study period | 55.2% vs 48.9% vs 16.8% |
|  | Murray (8) | 2018 | 1998 – 2012 | HESD | All ages | 1-year | (40.5% + 14.5%) * |
|  | Riall (9) | 2010 | 1996 – 2005 | Medicare | ≥ 66 years | 2-years | 38% * |

*PGBD, Percutaneous Gallbladder Drainage. SC, Supportive Care. RCT, Randomized Controlled Trial. NRD, Nationwide Readmissions Database, USA. HESD, Hospital Episode Statistics Database, England. *, Patients without an immediate cholecystectomy (recurrence + biliary-related complications).*

A PubMed search on readmissions in patients with acute cholecystitis according to the index treatment modality, obtained one randomized controlled trial,(1) three single-center studies,(3,5,6) and five studies from large administrative databases.(2,4,7–9) Half of the studies included patients of all ages, whereas only one study focused on octogenarians.(7) The follow-up period ranged from 30 days to 2 years. Readmission rate ranged from 6.4% (at 90 days) to 16.8% (encompassing the entire study period) in patients undergoing surgery during the index admission. On the other hand, readmission rate ranged from 32.7% (at 30 days) to 65% (at 1 year) in patients who received supportive care or percutaneous gallbladder drainage.

**References**

1. Loozen CS, van Santvoort HC, van Duijvendijk P, Besselink MG, Gouma DJ, Nieuwenhuijzen GA, et al. Laparoscopic cholecystectomy versus percutaneous catheter drainage for acute cholecystitis in high risk patients (CHOCOLATE): multicentre randomised clinical trial. BMJ. 2018 Oct 8;363:k3965.

2. Sanaiha Y, Juo YY, Rudasill SE, Jaman R, Sareh S, de Virgilio C, et al. Percutaneous cholecystostomy for grade III acute cholecystitis is associated with worse outcomes. Am J Surg. 2020;220(1):197–202.

3. Garcés-Albir M, Martín-Gorgojo V, Perdomo R, Molina-Rodríguez JL, Muñoz-Forner E, Dorcaratto D, et al. Acute cholecystitis in elderly and high-risk surgical patients: is percutaneous cholecystostomy preferable to emergency cholecystectomy? Journal of Gastrointestinal Surgery. 2020;24(11):2579–86.

4. Fleming MM, DeWane MP, Luo J, Liu F, Zhang Y, Pei KY. A propensity score matched comparison of readmissions and cost of laparoscopic cholecystectomy vs percutaneous cholecystostomy for acute cholecystitis. Am J Surg. 2019;217(1):83–9.

5. Kivivuori A, Salminen P, Ukkonen M, Ilves I, Vihervaara H, Zalevskaja K, et al. Laparoscopic cholecystectomy versus antibiotic therapy for acute cholecystitis in patients over 75 years: Randomized clinical trial and retrospective cohort study. Scand J Surg. 2023 Dec;112(4):219–26.

6. Turiño SY, Shabanzadeh DM, Eichen NM, Jørgensen SL, Sørensen LT, Jørgensen LN. Percutaneous Cholecystostomy Versus Conservative Treatment for Acute Cholecystitis: a Cohort Study. Journal of Gastrointestinal Surgery. 2019;23(2):297–303.

7. Wiggins T, Markar SR, Mackenzie H, Jamel S, Askari A, Faiz O, et al. Evolution in the management of acute cholecystitis in the elderly: population-based cohort study. Surg Endosc. 2018;32(10):4078–86.

8. Murray AC, Markar S, Mackenzie H, Baser O, Wiggins T, Askari A, et al. An observational study of the timing of surgery, use of laparoscopy and outcomes for acute cholecystitis in the USA and UK. Surg Endosc. 2018;32(7):3055–63.

9. Riall TS, Zhang D, Townsend CM, Kuo YF, Goodwin JS. Failure to Perform Cholecystectomy for Acute Cholecystitis in Elderly Patients Is Associated with Increased Morbidity, Mortality, and Cost. J Am Coll Surg. 2010;210(5):668–77.
